# Inaccurate Ascertainment of Morbidity and Mortality due to Influenza in Administrative Databases: A Population-Based Record Linkage Study

**DOI:** 10.1371/journal.pone.0098446

**Published:** 2014-05-29

**Authors:** David J. Muscatello, Janaki Amin, C. Raina MacIntyre, Anthony T. Newall, William D. Rawlinson, Vitali Sintchenko, Robin Gilmour, Sarah Thackway

**Affiliations:** 1 Centre for Epidemiology and Evidence, New South Wales Ministry of Health, North Sydney, W, Australia; 2 School of Public Health and Community Medicine, The University of New South Wales, Kensington, New South Wales, Australia; 3 The Kirby Institute, The University of New South Wales, Coogee, New South Wales, Australia; 4 South East Area Laboratory Service, The Prince of Wales Hospital, Randwick, New South Wales, Australia; 5 Faculty of Medicine, The University of New South Wales, New South Wales, Australia; 6 Sydney Medical School, The University of Sydney, Camperdown, New South Wales, Australia; 7 Centre for Infectious Diseases and Microbiology, Pathology West – Institute for Clinical Pathology and Medical Research, Westmead, New South Wales, Australia; The Australian National University, Australia

## Abstract

**Background:**

Historically, counting influenza recorded in administrative health outcome databases has been considered insufficient to estimate influenza attributable morbidity and mortality in populations. We used database record linkage to evaluate whether modern databases have similar limitations.

**Methods:**

Person-level records were linked across databases of laboratory notified influenza, emergency department (ED) presentations, hospital admissions and death registrations, from the population (∼6.9 million) of New South Wales (NSW), Australia, 2005 to 2008.

**Results:**

There were 2568 virologically diagnosed influenza infections notified. Among those, 25% of 40 who died, 49% of 1451 with a hospital admission and 7% of 1742 with an ED presentation had influenza recorded on the respective database record. Compared with persons aged ≥65 years and residents of regional and remote areas, respectively, children and residents of major cities were more likely to have influenza coded on their admission record. Compared with older persons and admitted patients, respectively, working age persons and non-admitted persons were more likely to have influenza coded on their ED record. On both ED and admission records, persons with influenza type A infection were more likely than those with type B infection to have influenza coded. Among death registrations, hospital admissions and ED presentations with influenza recorded as a cause of illness, 15%, 28% and 1.4%, respectively, also had laboratory notified influenza. Time trends in counts of influenza recorded on the ED, admission and death databases reflected the trend in counts of virologically diagnosed influenza.

**Conclusions:**

A minority of the death, hospital admission and ED records for persons with a virologically diagnosed influenza infection identified influenza as a cause of illness. Few database records with influenza recorded as a cause had laboratory confirmation. The databases have limited value for estimating incidence of influenza outcomes, but can be used for monitoring variation in incidence over time.

## Introduction

Influenza, an acute viral disease of the respiratory tract, is a major threat to public health. Antigenic shift, an introduction to human populations of avian or swine influenza type A viruses that are antigenically distinct from previous human influenza viruses, and increased human population mobility and air travel, create an unpredictable risk for rapid pandemic spread of influenza through global populations [1]–[4]. Despite vaccination campaigns, annual seasonal influenza type A and B epidemics also cause substantial burden of illness, death and cost to society [5].

Under-ascertainment of severe outcomes of influenza infections in administrative databases of health outcomes has long been recognised. After reviewing registered deaths in the London vital statistics register following an influenza epidemic in 1847, William Farr, commented [6]:

“…the epidemic carried off more than 5,000 souls over and above the mortality of the season… the deaths referred to that cause [influenza] are only 1,157”.

Ambiguity of influenza's role in illness recorded in databases of health outcomes arises from its lack of pathognomonic features, that is, lack of characteristic symptoms. Nevertheless, certain influenza-like symptoms such as fever with cough and fatigue are reasonable predictors of uncomplicated influenza infection particularly during epidemic influenza activity. On the other hand, many pathogens can cause influenza-like symptoms. Further, many patients present for medical care because of complications following an influenza infection, which can range from primary viral to secondary bacterial pneumonia, through pulmonary super-infections, exacerbations of chronic respiratory conditions, and even non-respiratory complications such as neurological complications, direct cardiac complications, or worsening of underlying cardiac conditions. Laboratory diagnosis is required to confirm influenza's role in infection, but this is not always clinically useful and the time window for successful detection is limited [1], [7]–[12].

For these reasons, specialist influenza epidemiologists are familiar with the need to estimate ‘excess’ morbidity and mortality attributable to influenza rather than using direct counting of influenza diagnoses on health outcomes databases. The term ‘excess’ is used because advanced statistical methods, typically time series analysis techniques, are required to estimate influenza's contribution to counts or population rates of broad health outcome categories such as pneumonia and influenza combined, all respiratory illness, respiratory and circulatory illness, or all-cause outcomes [13]–[16]. Similar methods are used in prospective public health surveillance of influenza-associated mortality [17]–[19].

The statistical methods used for estimating the incidence and burden of influenza outcomes can be difficult to understand, and results can be imprecise and vary according to the method used [14]. Such studies never allow the researcher to determine which of the subjects in the databases had an influenza infection. The approach is thus an indirect means of studying the epidemiology of influenza. In the authors' experience, this limits awareness, understanding and acceptance of the approach by non-specialist decision makers, policy makers and the general public. The resulting mortality estimates have been criticised as being over-estimated and inconsistent, leading to over-emphasis on influenza as a public health problem [20]. There are also several examples of databases being directly used to assess the population burden and epidemiology of influenza without accounting for incomplete ascertainment [21]–[25]. Further, two studies, one from a single hospital in a major city in New Zealand and one including all Western Australian children, may be interpreted to suggest that hospital discharge databases are quite useful for estimating the burden of hospitalised influenza because they reported quite high sensitivities up to 86% for hospital discharge diagnosis codes in identifying influenza infections that had been virologically confirmed by a pathology laboratory [26], [27].

To provide a comprehensive evaluation of the capacity of modern health outcome databases to accurately ascertain outcomes of influenza infection in a population, we used person-level record linkage of laboratory notified influenza, emergency department, hospital admission and death databases from the most populous (6.9 million) Australian state, New South Wales (NSW). NSW comprises approximately one third of Australia's population. There were four aims:

1. What proportion of persons with a virologically confirmed influenza infection had influenza recorded as a cause of illness on any corresponding emergency department (ED), hospital admission or death database records?
2. What factors were associated with the infection being recorded as a cause of illness on the database record?
3. What proportion of database records with influenza recorded as a cause of illness had an influenza infection diagnosed by a pathology service provider?
4. Do time trends of database records with influenza recorded as a cause of illness reflect time trends of pathology-diagnosed influenza?

## Materials and Methods

### Ethics statement

The project was approved by the NSW Population and Health Services Research Ethics Committee (HREC/09/CIPHS/41). Informed written consent of patients was not required because the record linkage used a privacy preserving method [28] and only anonymized, non-identifiable records were provided for analysis.

### Definitions

#### Laboratory notified influenza

Under NSW public health legislation, an influenza diagnosis made by a pathology service provider is a reportable (scheduled) medical condition. Laboratories are required to report the diagnoses to regional public health units who record the information in the Notifiable Conditions Information Management System database. Point of care tests are only included if performed in a laboratory.

#### Virological notification

Laboratory notified influenza diagnosed by virological methods, identified by test type: nucleic acid testing, antigen testing (including point-of-care testing) or culture; or specimen type: swab, sputum or lavage.

#### Serological notification

Laboratory notified influenza diagnosed by detecting influenza antibodies in a blood specimen, identified by test type: serology; or specimen type: ‘blood’ or ‘serum’.

#### Coded influenza

A coded diagnosis of influenza, including influenza with pneumonia, recorded in any of the emergency department, admitted patient or vital statistics (cause of death) databases using a disease classification.

#### Coded confirmed influenza

The code used in the database was from the International Classification of Diseases, Revision 10 (ICD-10) code groups J09 or J10 – Influenza due to an identified influenza virus.

#### Coded non-confirmed influenza

The code was from the ICD-10 code group J11 – Influenza, virus not identified.

#### Certified influenza death

Since coded deaths were only available 2005 to 2007, we used un-coded cause of death text from the death registration database to identify influenza in any part of the medically certified underlying or contributing causes of death. Some records may not have had cause of death information if they were referred to a coroner. Influenza was identified in the text using a previously published procedure [18].

#### Any-cause ED presentation, hospital admission or death registration

A database record which was not restricted according to coded diagnosis or cause of death.

### Record linkage

**Figure 1:**
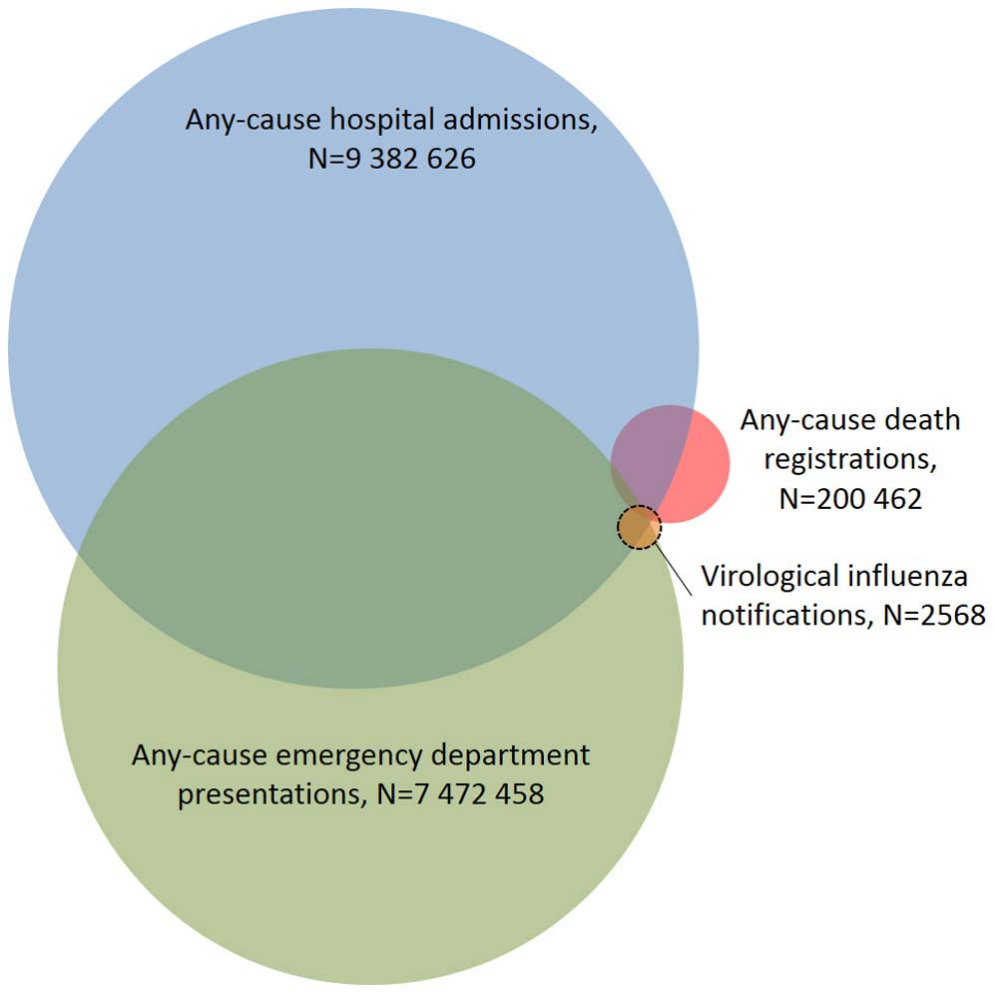
Venn diagram schematically showing the linked dataset, indicated by a dashed line, that was used to assess whether persons with a virological notification of influenza had influenza recorded as a cause of illness on their death, admission or ED presentation record, if any. Notes: 1. The size of each circle and degree of overlap are illustrative only and not drawn to scale. 2. Only laboratory results within ±84 days of a death, and within ±28 days of an admissions or ED presentation were included.

**Figure 2:**
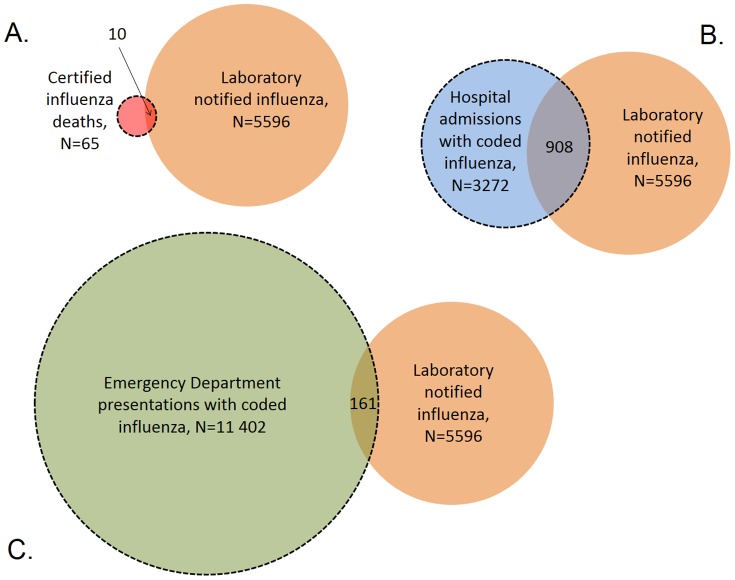
Venn diagrams schematically showing the linked datasets, indicated by dashed lines, used for assessing whether influenza recorded on the databases was based on a laboratory finding. The datasets included persons with A. certified influenza deaths; B. coded influenza hospital admissions; and C. coded influenza ED presentations and any notification (serological or virological) records for the same persons. Notes: 1. The size of each circle and degree of overlap are illustrative only and not drawn to scale. 2. Only laboratory results within ±84 days of a death, and within ±28 days of an admissions or ED presentation were included.

Privacy preserving, probabilistic record linkage was provided by the Centre for Health Record Linkage [28], [29]. For calendar years 2005 to 2008 we obtained laboratory notified influenza records in the NSW Notifiable Conditions Information Management System and their person-based intersection with the following NSW databases: the NSW Emergency Department (ED) Data Collection; the NSW Admitted Patient Data Collection, and deaths from the vital statistics register of the NSW Registry of Births, Deaths and Marriages (Figure 1). That linkage was used to achieve aims one and two. To achieve aims three and four, we also obtained records of all certified influenza deaths, and all coded influenza records from the ED and hospital admissions databases and their intersection with the notifications database (Figure 2).

The hospital admissions database included all NSW public and private hospital admissions. The database is equivalent to databases termed ‘hospital discharge’ databases in other studies. The information is derived from hospital patient administration information systems. Diagnoses are coded by health information managers or clinical coders in medical record departments at some time after the patient is discharged.

The ED (or ‘emergency room’ or ‘casualty’) database is derived from ED patient management information systems. Diagnoses are generally recorded in real-time on discharge from the ED, or entered shortly afterwards by either clinical or clerical staff. Some smaller regional hospitals did not participate in the ED Data Collection during the study period. The database included approximately 82% of all NSW public hospital ED presentations during the study period. Private hospitals accounted for only 2% of NSW ED presentations in 2008–2009 [30] and data for these were not available. In the Australian health system, the ED is almost always a pathway for patients requiring admission for acute illness, thus there is substantial overlap between the ED and admitted patient databases, as indicated in Figure 1.

### Classifications used

For 2005 to 2007 only, the registered death database included coded underlying and contributing causes of death from the national statistics bureau using the International Classification of Diseases, Revision 10 (ICD-10).The hospital admission database included a primary admission diagnosis and up to 50 additional diagnoses coded with the ICD-10, Australian Clinical Modification. Depending on the information system used at the hospital, the ED database included up to two provisional ED diagnoses coded using: ICD Revision 9 (ICD-9), ICD-10, or the Systematized Nomenclature of Medicine – Clinical Terminology (SNOMED-CT). The ICD-9 codes for influenza included the 487 code group, ICD-10 included J09-J11, and the SNOMED CT concepts used for influenza are listed in Table S2 in File S1. Only the first recorded ED diagnosis was used, as additional diagnoses are not mandatorily recorded.

Geographic remoteness of patients' statistical local area of residence was classified according to the Australian Standard Geographic Classification [31]. Co-morbidities among patients admitted to hospital were classified according to the Quan modification of the Charlson comorbidity index [32]. We used the primary and all other available admission diagnosis codes in assigning the comorbidity index.

### Inclusion criteria and linked datasets used

In the notification records, the patient pathology specimen collection date provides the best approximation to the date of onset of a confirmed infection, reflecting the date the patient interacted with a medical service and a clinical specimen was collected. Record linkage matches all events for the same person during the study period. Since influenza is an acute infection, we only included in our analysis ED presentations and hospital admissions within ±28 days, and deaths within ±84 days, of the specimen collection date of the laboratory notified influenza record. The longer window for deaths was used because influenza is increasingly recognised as a complicating or triggering factor in illnesses other than respiratory illness, such as cardiac disease. This could lead to an extended course of illness and delayed death [11]. There is also evidence that the duration of infection is lengthened in immunocompromised persons, who may be more likely to have a fatal illness [11], [33].

Only pathology service providers (laboratories) were eligible to notify influenza; notifications from all other sources were excluded. Duplicate notifications, or a second notification for the same person occurring within 84 days following the first, were excluded. Among multiple notifications, the earliest was chosen.

If there were multiple hospital admissions within the ±28 day window, a single admission was chosen as follows, in order of priority: the hospital stay period containing the specimen date of the notification; the hospital admission date closest to the specimen date; or the earliest admission. The same criteria were applied to ED presentations, based on the ED stay dates.

Aims one and two involved evaluation of the causes of illness recorded on the databases for persons with an influenza infection. Only virological notifications (Figure 1) were used in these analyses. This is because serological laboratory diagnosis may not accurately reflect an influenza infection. Definitive laboratory evidence for serological diagnosis requires paired sera collected during the acute and convalescent phases of infection [34]. Seroconversion in response to influenza vaccination is defined in the same way [35], and recent vaccination can therefore lead to false positive diagnosis. For serological notifications, the laboratory database did not include diagnostic criteria and thus we could not assess the integrity of the diagnosis. Consultation with Public Health Units that record the notifications in our state confirmed that few were based on paired results.

For aim three, involving assessment of whether influenza illness recorded on the databases was based on laboratory diagnosis, we used all laboratory notified influenza including serological notifications (Figure 2). Regardless of whether a serological result accurately represents an influenza infection, it does represent a positive pathology finding in response to a request from a clinician and is unlikely to be questioned when a death is certified, or an admission or ED presentation is coded.

For aim four, involving comparison of time trends of influenza recorded on the databases with time trends in the incidence of influenza infections, the same datasets as for aim three and Figure 2 were used except only virological notifications were included. This was to ensure that the time trends in certified influenza deaths and coded influenza were compared with the incidence of the more accurate virologically diagnosed influenza infections.

SAS version 9.3 in SAS Enterprise Guide version 5.1 was used for all data analysis. Logistic regression was used for aim 2; to assess factors independently associated with virological notifications having influenza recorded as a cause of illness in the databases.

## Results

After deleting duplicate and non-laboratory notifications, 5596 of the 5792 laboratory notified influenza records remained, of which 2568 (46%) were virological notifications.

The record linkage shown in Figure 1 identified that 28% of persons with a virological notification did not have an associated ED presentation, admission or death, suggesting those specimens were collected in community-level care. More than two thirds (68%) of persons with a virological notification had an associated any-cause ED presentation, 57% had an any-cause hospital admission, and 1.6% died. Just over one half (52%) of the virological notifications were in persons with both a hospital admission and ED presentation (Table S1 in File S1).

Among persons with a virological notification and an any-cause death, the median absolute value of the time interval from specimen collection date to death was 16 days (lower quartile  =  five days, upper quartile  = 32 days) indicating that one half of the deaths occurred within 16 days and three quarters within 32 days of specimen collection. For those with an any-cause admission or ED presentation, the specimen date was much more closely clustered around the admission and presentation date. In both cases, the median absolute value of the interval was one day (lower quartile  =  zero days, upper quartile  =  two days).

The distributions of the raw (non-absolute) values of the specimen collection date to outcome intervals are shown graphically in Figure S1 in File S1. Among persons who died, the majority of the specimen dates were between zero and 28 days prior (range 79 days prior to five days after) the date of death. For admissions, the highest frequency of specimen collection was day one after admission, with the majority collected from zero to two days after. Few were collected prior to admission. For ED presentations, the pattern was similar to that of admissions, although day zero was more prominent than in admissions.

Among patients with a virological notification during our study period, 88 hospitals were represented among the patients that had a hospital admission. There were 70 hospitals represented among the patients that had an ED presentation. Of those 88 and 70 hospitals, all but four hospitals receive patients of any age. The remaining four include two solely paediatric and two solely adult hospitals, all of which are in the NSW capital city, Sydney. The two children's hospitals account for 3% of both total hospital admissions and ED presentations in the state [36], but accounted for 27% and 23% of the virological notifications in our study with admissions and ED presentations, respectively.

**Figure 3:**
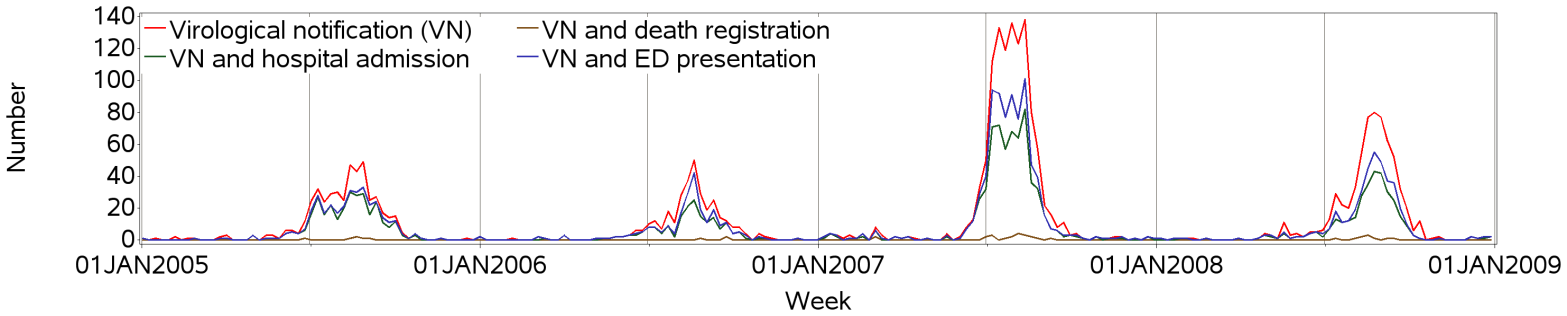
Time series of weekly counts of virological notifications and of those with an any-cause death registration, hospital admission or emergency department (ED) presentation, NSW, Australia, 2005–2008.

Figure 3 shows the epidemic patterns in the time series of virological notifications, and of those with an any-cause death, hospital admission or emergency presentation. The seasonal influenza in 2007 clearly represents the largest epidemic of the years studied.

### Descriptive comparison of the virological notifications with corresponding any-cause ED presentations, hospital admissions and deaths, and with influenza recorded on the databases

In 2008, children aged <15 years comprised 19% and persons aged ≥65 years comprised 14% of the NSW population and females comprised 51% [37]. In comparison, as shown in Table 1, a much larger proportion of persons with a virological notification (56%) was aged <15 years. ED presentations with coded influenza showed a more evenly spread age distribution. Hospital admissions with coded influenza were skewed towards younger persons but not to the extent of virological notifications. Females (46%) were somewhat under-represented among persons with virological notifications (Table 1).

**Table 1:** Descriptive comparison of all virological notifications (VN), those with an any-cause ED presentation, hospital admission or death registration, and of coded influenza and certified influenza deaths in the databases, New South Wales, Australia, 2005–2008.

	Virological notification (VN) N = 2,568	VN and any-cause ED presentation N = 1,742	ED presentations with coded influenza N = 11,402	VN and any-cause hospital admission N = 1,451	Hospital admissions with coded influenza^1,2^ N = 3,272	VN and any cause death N = 40	Certified influenzadeaths^3^ N = 65
Category	%	%	%	%	%	%	%
Influenza recorded as a cause of illness^4^	100.0	6.8	100.0	48.9	100.0	25.0	100.0
Age (years)							
<15	55.5	65.2	21.9	68.1	40.8	5.0	1.5
15–64	33.5	25.1	71.0	19.4	42.2	27.5	12.3
≥65	11.0	9.8	7.2	12.5	17.0	67.5	86.2
**Total**	**100.0**	**100.0**	**100.0**	**100.0**	**100.0**	**100.0**	**100.0**
Sex							
Male	53.7	53.7	48.9	53.9	48.9	57.5	38.5
Female	46.3	46.3	51.1	46.1	51.1	42.5	61.5
**Total**	**100.0**	**100.0**	**100.0**	**100.0**	**100.0**	**100.0**	**100.0**
Geographic remoteness							
Major city	70.4	78.2	50.6	81.3	62.9	72.5	50.8
Regional, remote	28.3	20.9	45.6	18.0	33.3	27.5	47.7
Non-NSW resident or not classifiable	1.3	0.9	3.9	0.8	3.8	0.0	1.5
**Total**	**100.0**	**100·0**	**100.0**	**100.0**	**100.0**	**100.0**	**100.0**
Year							
2005	18.1	19.1	24.4	21.4	23.7	15.0	21.5
2006	12.9	13.2	18.9	12.8	16.5	7.5	7.7
2007	43.7	45.0	33.2	43.4	36.6	52.5	40.0
2008	25.4	22.7	23.6	22.5	23.1	25.0	30.8
**Total**	**100.0**	**100.0**	**100.0**	**100.0**	**100.0**	**100.0**	**100.0**
Virus type							
Influenza A	70.0	70.7	Na	69.9	Na	77.5	Na
Influenza B	28.0	27.6	Na	28.3	Na	22.5	Na
Influenza A&B	0.2	0.2	Na	0.2	Na	0.0	Na
Not recorded	1.9	1.6	Na	1.7	Na	0.0	Na
**Total**	**100.0**	**100.0**	**Na**	**100.0**	**Na**	**100.0**	**Na**

Notes:

na  =  not applicable.

1. Two hospital admission records with coded influenza had missing age and sex.

2. Coded influenza admissions were based on all 51 available admission diagnosis fields.

3. Two registered deaths had no cause recorded.

4. Coded influenza in the primary ED diagnosis in the ED database, or in any available admission diagnosis in the admission database, or influenza certified as a cause of death in the death database.

In 2008, approximately one quarter (26%) of the NSW population resided in regional or remote areas [31], [37], [38]. In comparison, almost one half of patients with coded influenza in their ED presentation record (46%) and with a certified influenza death (48%), and one third of patients with coded influenza in their hospital admission record resided in a regional or remote area (Table 1).

Seventy per cent of persons with a virological notification had an influenza type A infection. The proportion was similar among those with an any-cause ED presentation or hospital admission. A higher proportion (78%) of those with an any-cause registered death was influenza type A (Table 1).

In 2008, persons aged <15 years comprised 1.2% and those aged ≥65 years comprised 82% of all deaths of NSW residents [37]. Persons with a virological notification and an any-cause registered death had a younger age distribution (68% aged ≥65 years). Persons with a certified influenza death had a slightly older age distribution with 86% aged ≥65 years (Table 1).

### What proportion of persons with a virological notification and an any-cause database record had influenza recorded as the cause of the illness?

Of the 40 persons during the study period who had a virological notification and who died within ±84 days of specimen collection, ten (25%) were influenza certified deaths (Table 1). For the 30 deaths with official cause of death coding (only available 2005 to 2007), six had coded influenza as the underlying cause. Other or unspecified pneumonia and neoplasms were recorded in 5 cases each. Circulatory illness was coded in 10 of 30 (Figure S2 in File S1). All coded influenza was recorded as an underlying cause – none had influenza coded as a contributing cause.

As a sensitivity analysis to account for the possibility of influenza not contributing to deaths more distal in time from the specimen date, we repeated the analysis with a 28 day window around the specimen date. There were 9 (31%) of 29 such deaths that had an influenza cause certified. We also reviewed the cause of death text on the 40 virological notifications with an any-cause death registration within 84 days. None were injuries. Two had no information, possibly because they were coronial cases and in each of those the interval between date of death and specimen collection was 1 day or less.

Of the 1451 persons during the study period who had a virological notification and an any-cause hospital admission within ±28 days of specimen collection, 709 (49%) had coded influenza in any admission diagnosis field (Table 1). Coded influenza was recorded as a primary diagnosis in 38%, other or unspecified pneumonia in 8.6% and other respiratory illness in 24%. The symptom fever or an unspecified infection was coded as a primary diagnosis in 9.0% (Figure S3 in File S1).

Among the 1742 persons with a virological notification and an any-cause ED presentation within ±28 days of specimen collection, 119 (6.8%) had coded influenza. Another 9.7% had other or unspecified pneumonia coded, and 29% had other respiratory illness. The symptom fever or an unspecified infection was coded in almost one quarter (23%). The ED diagnosis was missing in 189 (11%) of the ED records (Figure S4 in File S1).

### Factors associated with influenza being recorded as a cause of illness in the databases

Most likely due to the small number of persons with a virological notification and who died (40), we did not identify any association between having an influenza certified death and the variables age, sex, remoteness of residence, or influenza type.

There were 1414 persons with a virological notification and an any-cause hospital admission who resided in NSW and whose address could be classified by remoteness, and who had non-missing values of other variables. Among these persons, factors that were independently and positively associated with having coded influenza in any diagnosis field on the admission record after controlling for sex, length of hospital stay and co-morbidities were: being aged <15 years compared with being aged ≥65 years, having an influenza A infection compared with influenza B, and residing in a major city compared with residing in regional and remote areas (Table 2).

**Table 2:** Factors^1^ independently associated with having coded influenza^2^ in any diagnosis on the admission record among persons with an any-cause hospital admission and a virological notification, New South Wales, 2005 to 2008.

Category	Virological notification and coded influenza (N = 1414)^3,4^ Number (%)	Adjusted odds ratio^5^	95% confidence limits		p-value
Age (years)					
**<15**	**492 (34.8)**	**1.509**	**1.047**	**2.175**	**0.0275**
15–64	126 (8.9)	1.266	0.848	1.891	0.2490
≥65	73 (5.2)	Reference category			
Sex					
Male	380 (26.9)	Reference category			
Female	311 (22.0)	0.925	0.747	1.145	0.4721
Virus type					
** Influenza A**	**534 (37.8)**	**1.788**	**1.411**	**2.265**	**<.0001**
Influenza B	157 (11.1)	Reference			
Remoteness					
** Major city**	**593 (41.9)**	**1.541**	**1.160**	**2.048**	**0.0028**
Regional, remote	98 (6.9)	Reference category			
Length of stay					
Per day	Not applicable	1.006	0.993	1.019	0.3877
Co-morbidity index					
Per 1 unit increase	Not applicable	0.984	0.868	1.116	0.8038

Notes:

1. Statistically significant results are shown in bold.

2. Coded influenza in the admission database could include any of ICD-10 codes J09-J11.

3. Includes coded influenza in any of the available diagnosis fields on the admission record.

4. Of 1451 records, 37 (3%) records without an influenza type A or B result or for prison or non-NSW residents were excluded from the analysis.

5. Each variable was adjusted for all other variables in the table using logistic regression.

There were 1512 persons with a virological notification and any-cause ED presentation who resided in NSW and whose address could be classified by remoteness, and who had non-missing values of other variables. Factors that were independently and positively associated with having coded influenza on the ED record were: being aged 15–64 years compared with being older, having an influenza type A infection compared with influenza B, and being discharged home from emergency rather than being admitted to hospital. Residence in a major city showed a negative association with borderline statistical significance compared with residence in regional or remote areas (Table 3). ED triage urgency category and arrival by ambulance were not associated with having coded influenza and were excluded from the final model.

**Table 3:** Logistic regression analysis of factors^1^ independently associated with having coded influenza^2^ in the ED provisional diagnosis among persons with an any-cause ED presentation and a virological notification, New South Wales, 2005 to 2008.

Category	Virological notifications and coded influenza (N = 1512)^3^ Number (%)	Adjusted odds ratio^4^	95% confidence limits		p-value
Age (years)					
<15	45 (3.0)	0.739	0.304	1.798	0.5050
** 15–64**	**65 (4.3)**	**2.524**	**1.027**	**6.202**	**0.0436**
≥65	6 (0.4)	Reference			
Sex					
Male	64 (4.2)	Reference			
Female	52 (3.4)	0.774	0.516	1.160	0.2146
Virus type					
** Influenza A**	**92 (6.1)**	**1.688**	**1.038**	**2.746**	**0.0348**
Influenza B	24 (1.6)	Reference			
Remoteness					
Major city	67 (4.4)	0.651	0.420	1.007	0.0537
Regional, remote	49 (3.2)	Reference			
Discharge status					
** Discharged from ED**	**80 (5.3)**	**3.815**	**2.457**	**5.925**	**<.0001**
Admitted	36 (2.4)	Reference			

Notes:

1. Statistically significant results are shown in bold.

2. Coded influenza in the ED database could include ICD-9 codes (487) or ICD-10 codes (J09-J11) for influenza, or equivalent SNOMED-CT concepts (see File S1).

3. Of 1742 records, 230 (13%) records did not have a diagnosis recorded, did not have an influenza type A or B result, or did not have an address that could be classified according to remoteness, and were excluded from the analysis.

4. Each variable was adjusted for all other variables in the table using logistic regression.

### Do persons with influenza recorded on the database as a cause of illness also have a pathology finding indicating an influenza infection?

As indicated in the methods, these results are based on datasets obtained by record linkage of all 5596 notifications, either serological or virological, with certified influenza deaths, coded influenza hospital admissions and coded influenza ED presentations (Figure 2). Of the 65 certified influenza deaths during 2005 to 2008, ten (15%) also had laboratory notified influenza (Figure 2A). Among the 3272 influenza coded hospital admissions during the same period, 908 (28%) also had laboratory notified influenza (Figure 2B). Of the 11402 ED presentations with coded influenza, 161 (1.4%) had laboratory notified influenza (Figure 2C).

### Inconsistencies between coding and laboratory notification in hospital admission records

Of 2364 influenza coded admissions, 588 (25%) had coded confirmed influenza but no laboratory notified influenza record (serological or virological). Of 908 influenza coded hospital admissions that also had a laboratory-notified influenza record, 119 (13%) had coded non-confirmed influenza.

### Do the time series of certified influenza deaths and coded influenza reflect trends in time series of virological notifications?

**Figure 4:**
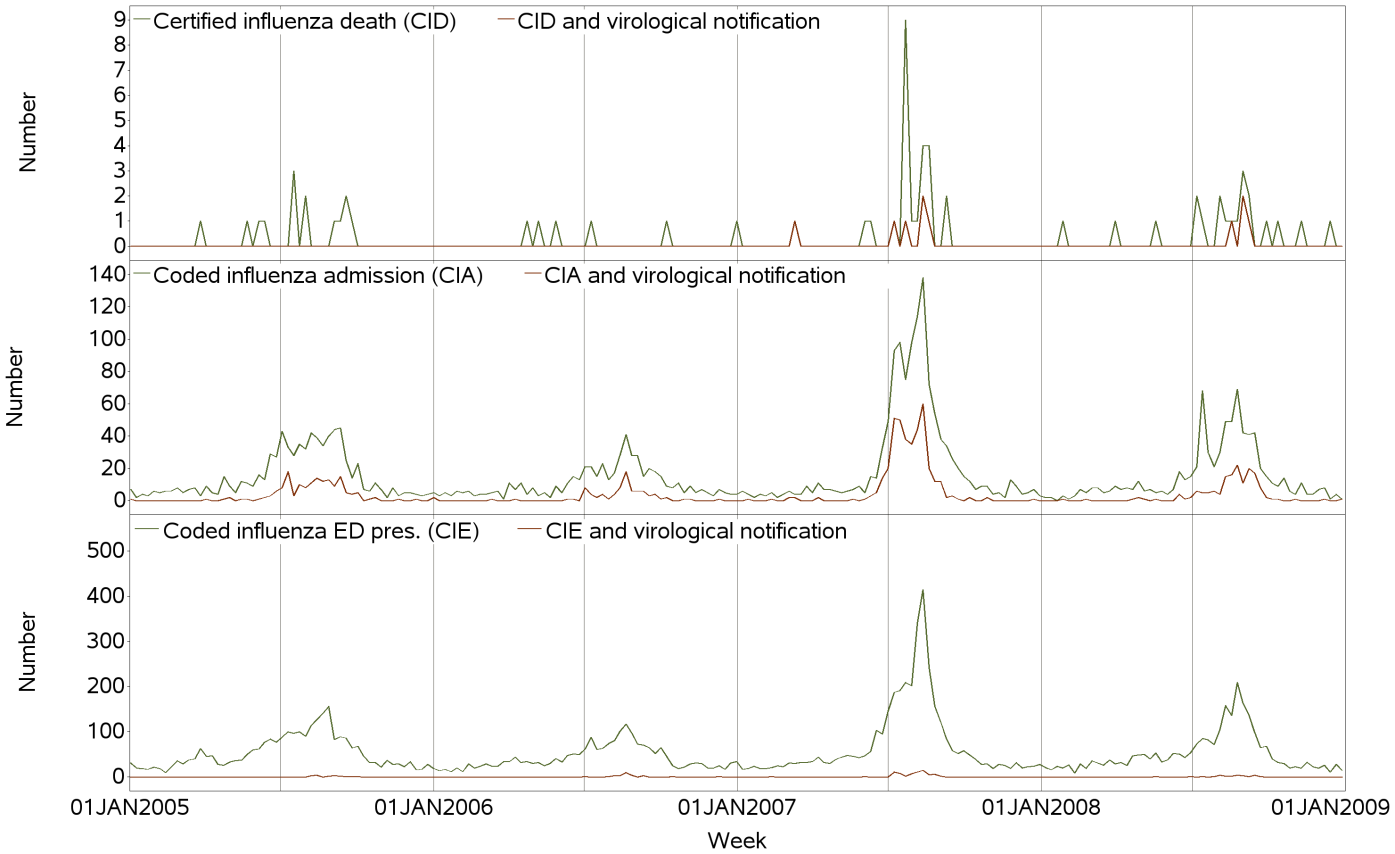
Time series of weekly counts of persons with: emergency department (ED) presentations with coded influenza; hospital admissions with coded influenza; influenza certified deaths; and those that also had a virological notification, New South Wales, Australia, 2005–2008.

Figure 4 shows the time series of weekly counts of certified influenza deaths and coded influenza from the hospital and ED databases, and the subset of each with a virological notification. Periods of markedly increased incidence of coded influenza and certified influenza deaths and the subset with virological notifications clearly correspond to epidemic periods evident in Figure 3.

## Discussion

In this comprehensive, population-based study evaluating three health outcomes databases capturing relatively mild to fatal illness, we found that nearly three quarters of virological notifications were from patients who had hospital ED or admission encounters. Over half (57%) were from patients who were hospitalised. Compared with the age distribution of the general population and with the age distribution of patients with influenza recorded as a cause of illness in the databases, children were strongly over-represented among those notifications. Compared with the geographic distribution of the general population, persons from regional and remote areas were over-represented among persons with influenza recorded as a cause of illness on all three databases, regardless of their laboratory confirmation status.

Persons with a virologically diagnosed infection had influenza recorded as a cause of illness in their corresponding ED, hospital and death records less than half the time. Hospital admission records, coded after a patient is discharged from hospital, were most likely (49%) to identify influenza as a cause of the admission, using all available diagnosis fields. Death records (25%) and ED presentations (7%) were far less likely to identify influenza. Many deceased persons or patients with influenza will not have been tested. Therefore, we cannot estimate the sensitivity of the databases in identifying all ED presentations, admissions and deaths that were outcomes of influenza infection. Nevertheless, these results indicate the databases have low sensitivity for influenza infections that were virologically diagnosed.

Logistic regression analyses showed that, compared with persons aged ≥65 years, children with a virological notification were more likely than other age groups to have influenza coded on their admission record, whereas working age persons were more likely to have influenza coded on their ED record. Compared with residents of regional and remote areas, residents of major cities with a virological notification were more likely to have influenza coded on their admission record. Persons with a virological notification and an ED presentation were more likely to have influenza coded on their ED record if they were discharged from the ED rather than admitted to hospital. On both ED and admission records, persons with influenza type A infection were more likely than those with type B infection to have influenza coded.

Among database records with influenza recorded as a cause of illness, we found low proportions (15% for deaths; 28% for admissions, and 1.4% for ED) were supported by a laboratory diagnosis. Since laboratory diagnosis is required to confirm an influenza infection, this suggests the majority of the diagnoses were clinical and therefore of uncertain accuracy. As described in the methods, the accuracy of serological diagnoses made in our state is also uncertain even though they are notified under our surveillance case definition. Nevertheless, we included these in this part of our analysis because a serology result represents a test ordered in a clinical setting to diagnose an acute illness. Whether accurately or inaccurately, the pathology provider made a diagnosis of influenza and returned the result to the treating clinician. The result, if done in the hospital setting, would be included on the hospital medical record, and the coder would, in principle, consider that information when coding.

The over-representation of children and children's hospitals among the notifications can at least in part be explained by the children's hospitals' in the capital city, Sydney, having a policy of ordering a laboratory test for all children presenting with influenza-like illness. The relatively high proportion of persons with coded influenza or an influenza-certified death that were residents of regional and remote areas deserves further research. If this does reflect a higher incidence of infection it could signal an unmet need for preventive services. Distance to health services is an important factor in influenza vaccination uptake in older persons [39]. Access to primary health care is somewhat limited in regional Australia [40] and influenza vaccination uptake was found to be lower in rural areas [41]. Slow turnaround times for laboratory testing in more remote areas may also limit the use of pathology services.

The large proportion of laboratory notified influenza that corresponded to a hospital admission indicates that notified influenza over-represents severe infections. It is highly improbable that these infections were representative of the spectrum of illness across the whole population. Around one third of seasonal influenza infections in healthy adults are asymptomatic [42] and general practice consultations occur around 20 times more frequently than hospital admissions for influenza A infections [43]. The result probably reflects the lack of clinical value in testing patients with mild illness.

Our finding of low sensitivity of coded influenza in the hospital admission database for identifying virologically confirmed influenza is at odds with the two studies that each found reasonably high sensitivity (86%) when using multiple diagnosis fields to locate influenza diagnosis codes [26], [27]. One study was conducted in a single large city in New Zealand and the other considered only children in another Australian state. Our study considered an entire state population of persons of all ages and thus our findings may reflect regional and age-based variation in clinical and coding practice.

The association between confirmed influenza type A infection and coded influenza compared with influenza type B among patients in the ED and admission setting might be explained by differing clinical presentations of illness due to each type. Two factors would influence the coding of influenza in the ED and admitted patient settings; the diagnosis made and recorded by the clinician, and the availability of the laboratory result at the time of diagnosis or coding. Since the availability of the laboratory result would not depend on the influenza type, the clinical diagnosis is likely to be the important factor. Influenza type A infections may have a more classic influenza-like presentation than type B infections. In a systematic review of experimental virus challenge studies, Carrat et al [42] reported a lower incidence of fever for influenza type B versus type A. However, a systematic review of the burden of influenza B reported only limited differences between clinical presentation of type A and B infections, but symptom comparisons were only available from studies of influenza burden in children [44].

We identified some unexpected but substantial inconsistencies between the influenza ICD-10 code used in hospital admission records and the laboratory notification status of the patient, including use of J11 – unconfirmed influenza, for admissions with laboratory findings, and use of J10 – confirmed influenza, for admissions without notified laboratory findings. Reasons for coded confirmed influenza not having an associated laboratory record could include coding error, incomplete laboratory notification, or use of point of care influenza diagnostic tests without referral of specimens to a laboratory for confirmation. Reasons for coded non-confirmed influenza having an associated laboratory record could include coding error, the test being done in the community setting rather than in the hospital, or unavailability of the laboratory result at the time of coding. Regular, routine audit and quality improvement of both coding and notification completeness would provide greater confidence in the accuracy of databases.

The time series of influenza diagnoses from each database coincided with epidemic activity marked by notifications, albeit with less distinct epidemic boundaries. Given few of the ED presentations were based on laboratory diagnosis, the concurrence in trends means many of those patients may have actually had influenza. Compared with a gold standard virological test, clinician judgement showed a low sensitivity (29%) and high specificity (92%) for diagnosing influenza in adults [45]. While these ED diagnoses therefore underestimate the incidence of influenza presentations, the high specificity supports the use of rapidly collected ED influenza diagnoses for monitoring trends in epidemic influenza as already conducted in our state and elsewhere [46], [47]. This is also consistent with the ecological but statistically significant correlations between time series of these diagnoses in our state [48], [49]. Even though our study shows that confirmed influenza infections are unlikely to receive an influenza diagnosis in ED, the broader age spectrum and large number of the ED presentations coded with influenza suggests that these may be more representative of the epidemiology of at least milder influenza infections in the population than the other databases evaluated in this study. Systematic research that more directly evaluates this question would be valuable.

The relatively few deaths and lack of complete official cause of death coding for the entire study period, limited the analyses we could do of factors associated with influenza being recorded on the death record, and with use of specific ICD-10 codes relative to laboratory diagnosis status. Place of death, such as home, nursing home or hospital could be an important factor influencing whether a laboratory diagnosis occurs, but we did not have access to that information.

There were other limitations of our study. ED presentations were partially underestimated due to incomplete state coverage. Only 2% of influenza type A notifications had a more specific subtype recorded, which prevented strain-specific analysis. Infection severity was not available on the notifiable conditions database, preventing stratification of our results by disease severity. Delays in official cause of death coding and release of unit record files prevented analysis of certified influenza deaths for 2008. Registered death information was reported by year of registration rather than year of death and coronial inquests could lead to absence of cause of death information in some registrations.

A further limitation was that the broad time windows of ±28 days for ED presentations and admissions, and ±84 days for deaths, may have led to some outcomes being coincidental with the influenza infection. The extremely narrow distributions of ED presentations and admissions around the specimen collection date and the large proportion of respiratory, fever and infection diagnoses support influenza at least playing a contributing role in the majority of these outcomes. The distribution of deaths relative to the specimen date was broader, but the sensitivity analysis we conducted reducing the 84 day window to 28 days, did not appreciably alter our conclusions.

## Conclusions

In this large and comprehensive record linkage study, we found that children and persons with more severe influenza-related morbidity requiring hospital treatment were over-represented in the laboratory notified influenza database. The under-identification of confirmed influenza infections combined with the lack of laboratory confirmation of influenza-classified outcomes in the ED, admission and death databases makes it clear that the population incidence of influenza outcomes cannot be accurately ascertained from these databases. Our study does not support use of these databases, which are readily available and often used in the government sector, for representatively studying the epidemiology or burden of influenza in the population without accounting for incorrect ascertainment. However, such databases are nonetheless useful as an indicator of time trends for surveillance.

## Supporting Information

File S1 **Supplementary tables S1 and S2 and figures S1, S2, S3 and S4.**
(PDF)
