# Supplementary material for: Inaccurate Ascertainment of Morbidity and Mortality due to Influenza in Administrative Databases: A Population-Based Record Linkage Study
Source: PLoS One. 2014 May 29;9(5):e98446. doi: 10.1371/journal.pone.0098446 (PMC4038604; doi:10.1371/journal.pone.0098446)
Supplement: File S1 — Supplementary tables S1 and S2 and figures S1, S2, S3 and S4. (PDF) [file pone.0098446.s001.pdf]

## Supplementary file S1: Supplementary tables and figures

**Table S1. Record linkage results for Figure 1 of the main manuscript: number and proportion of virological notifications that coincided with an any-cause ED presentation, hospital admission, or death, New South Wales, 2005-2008**

| Record linkage result | N | %[3] |
|---|---|---|
| No ED, admission or death record | 711 | 27.7 |
| Any-cause ED presentation[1] | 1742 | 67.8 |
| Any-cause hospital admission[1] | 1451 | 56.5 |
| Any-cause ED presentation or admission[1] | 1851 | 72.1 |
| Any-cause ED presentation and admission[1] | 1342 | 52.3 |
| Death[2] | 40 | 1.6 |
| **Total** | **2568** | **100.0** |

Notes:

1. Within ±28 days of specimen collection
2. Within ±84 days of specimen collection
3. Percentages do not sum to 100% because notifications can fit into multiple categories

**Table S2. Systematized Nomenclature of Medicine Clinical Terminology (SNOMED CT) concepts identifiers used to select coded influenza in the emergency department database in hospitals reporting SNOMED CT diagnoses.**

| | | | |
|---|---|---|---|
| 1553003 | 120843002 | 166211003 | 359833009 |
| 2731000 | 120844008 | 192691003 | 367026003 |
| 6142004 | 120907007 | 192693000 | 367027007 |
| 24662006 | 120917002 | 194946005 | 367256002 |
| 28105000 | 120918007 | 195878008 | 367257006 |
| 41269000 | 120919004 | 195920000 | 389069008 |
| 42964004 | 120920005 | 195921001 | 389070009 |
| 43692000 | 120921009 | 195922008 | 406588006 |
| 46171006 | 120922002 | 195923003 | 407477006 |
| 55014007 | 120923007 | 195924009 | 407478001 |
| 55207002 | 120925000 | 195925005 | 407479009 |
| 55604004 | 121006005 | 195927002 | 407480007 |
| 61700007 | 121008006 | 195928007 | 407481006 |
| 63039003 | 121026009 | 195929004 | 407482004 |
| 74644004 | 122004005 | 195930009 | 408687004 |
| 78046005 | 122005006 | 195931008 | 420362005 |
| 78431007 | 122006007 | 195933006 | 420508007 |
| 81524006 | 122007003 | 195934000 | 421264001 |
| 83440004 | 122008008 | 196200002 | 421539000 |
| 84037004 | 122009000 | 196201003 | |
| 84113001 | 122010005 | 196202005 | |
| 84512003 | 122011009 | 196203000 | |
| 88823001 | 122012002 | 230188005 | |
| 95891005 | 122013007 | 243612002 | |
| 103516006 | 122262002 | 243613007 | |
| 103517002 | 122349003 | 243614001 | |
| 103518007 | 122350003 | 259856001 | |
| 103519004 | 134249005 | 260210008 | |
| 103520005 | 134251009 | 266353003 | |
| 103521009 | 139168000 | 266391003 | |
| 103522002 | 143574001 | 266393000 | |
| 103523007 | 143575000 | 274104008 | |
| 103524001 | 155548002 | 285342006 | |
| 104310006 | 155559006 | 309789002 | |
| 111876006 | 155560001 | 309806000 | |
| 117817000 | 155561002 | 313251006 | |
| 117865004 | 155562009 | 315142009 | |
| 118089005 | 155564005 | 315143004 | |
| 118090001 | 161913008 | 315642008 | |
| 120753009 | 166210002 | 359829002 | |

**Figure S1. Distribution of the number of days from the date of A. any-cause death (N=40), B. any-cause hospital admission (N=1451) and C. any-cause ED presentation (N=1742), to the date of the patient's virological specimen.** Negative values indicate the specimen was taken before the outcome occurred.

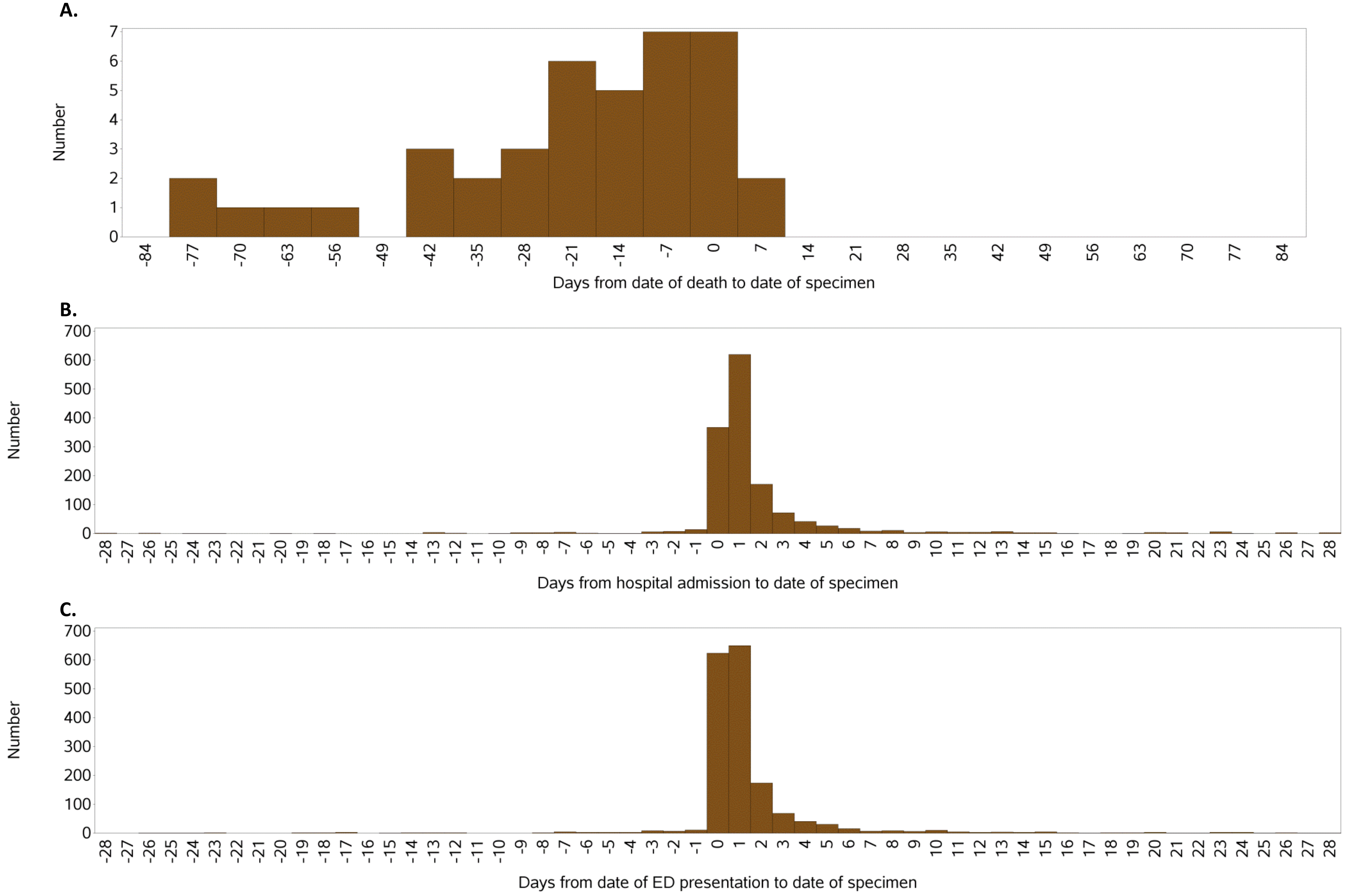

**Figure S2. Officially coded underlying cause of death of 30 persons with a virological notification and death within ±84 days of specimen collection, New South Wales, 2005-2007[1]**

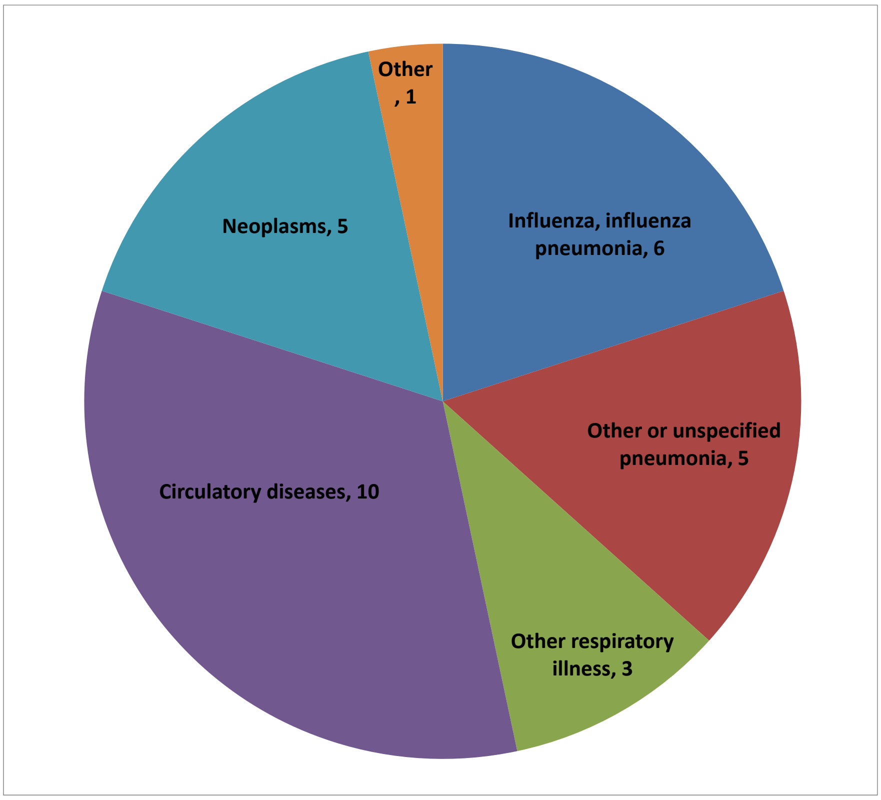


Notes:

1. Official cause of death coding was not available for 2008

ICD-10 codes used:

- Influenza or influenza pneumonia - J09-J11 (Influenza, including influenza with pneumonia).
- Other unspecified pneumonia – A20.2, A48.1, A70, B01.2, B05.2, B25.0, B59, J12-J18, J69, J85.1, P23, U04 (viral, bacterial or unspecified pneumonia due to specified or unspecified organisms other than influenza, pneumonitis due to solids and liquids, severe acute respiratory syndrome).
- Other respiratory illness - A37, all other J codes, P20-P28, R04.2-R06, R09.0-R09.3, R09.89 (all respiratory conditions other than pneumonia or influenza and including pertussis (whooping cough), cough symptom, respiratory distress or arrest and other breathing difficulties, shortness of breath or respiratory conditions during the perinatal period).
- Neoplasms – Chapter C (all cancers).
- Circulatory diseases – Chapter I (all circulatory conditions).

**Figure S3. Primary hospital admission diagnosis among 1452 persons with a virological notification and hospital admission within ±28 days of specimen collection, New South Wales, 2005-2008**

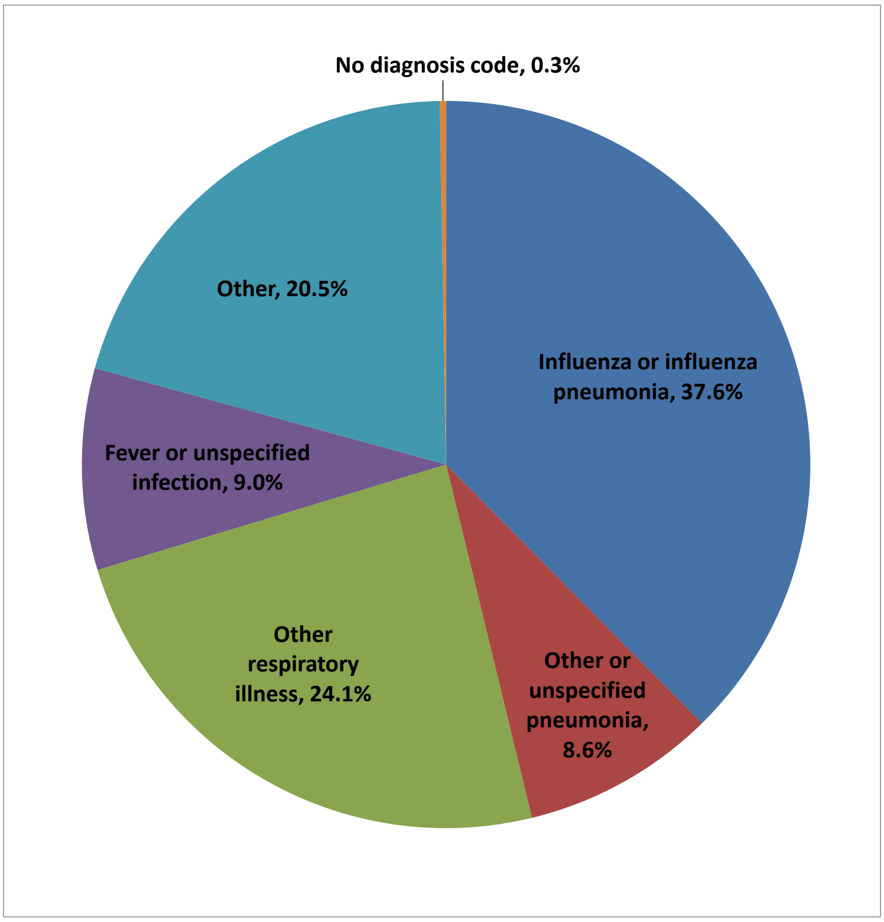


ICD-10 codes used:

- Influenza or influenza pneumonia - J09-J11 (Influenza, including influenza with pneumonia).
- Other unspecified pneumonia – A20.2, A48.1, A70, B01.2, B05.2, B25.0, B59, J12-J18, J69, J85.1, P23, U04 (pneumonia due to specified or unspecified organisms other than influenza, pneumonitis due to solids and liquids, severe acute respiratory syndrome).
- Other respiratory illness - A37, all other J codes, P20-P28, R04.2-R06, R09.0-R09.3, R09.89 (all respiratory conditions other than pneumonia or influenza and including pertussis (whooping cough), cough symptom, respiratory distress or arrest and other breathing difficulties, shortness of breath or respiratory conditions during the perinatal period).
- Fever or unspecified infection - A41.9, A49.9, A68, A99, B34.9, B97.8, B96.88, B99, R50 (fever symptom, unspecified viral infection, unspecified viraemia, unspecified bacteraemia, unspecified bacterial infection, unspecified infection).

**Figure S4. Primary emergency department provisional diagnosis among 1742 persons with a virological notification and emergency department presentation within ±28 days of specimen collection, New South Wales, 2005-2008**

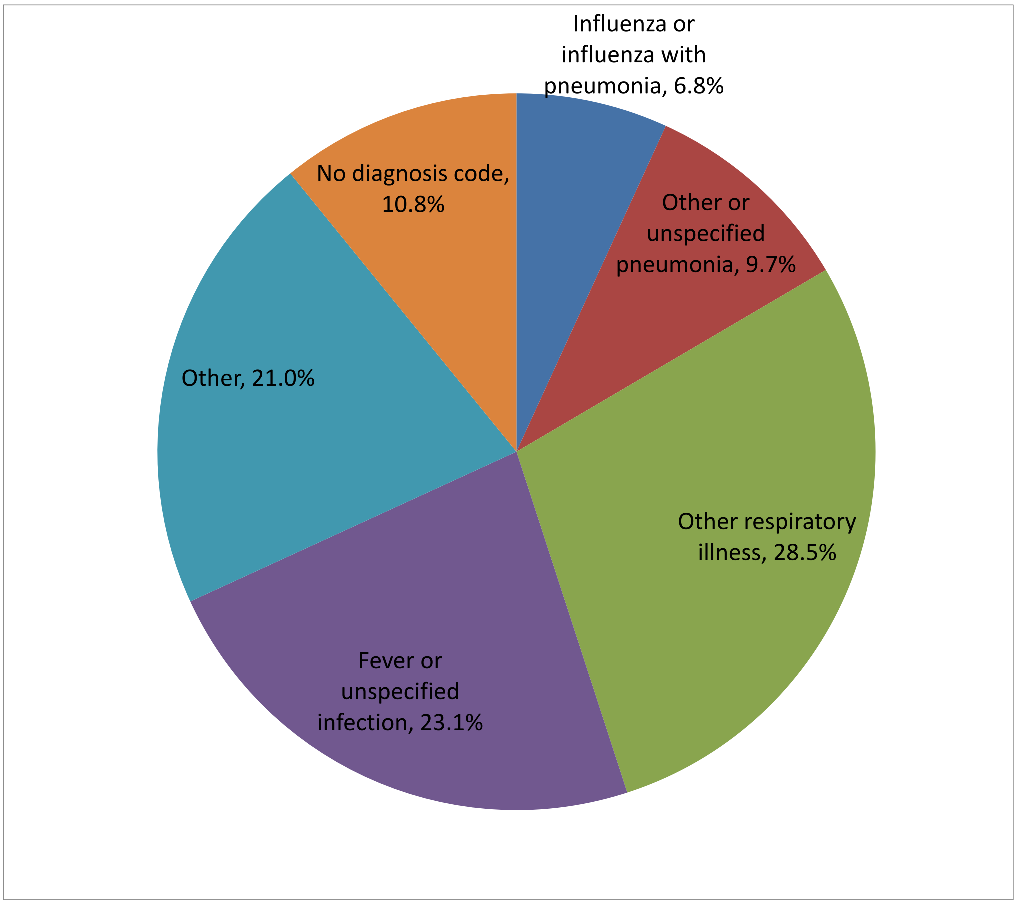


ICD-10 codes used (see Note below):

- Influenza or influenza pneumonia - J09-J11 (Influenza, including influenza with pneumonia).
- Other unspecified pneumonia – A48.1, J12-J18, U04 (non-influenza or unspecified pneumonia from Chapter J, legionella infection, severe acute respiratory syndrome).
- Other respiratory illness - A37, all other J codes, P20-P28, R04.2-R06, R09.0-R09.3, R09.89 (all respiratory conditions other than pneumonia or influenza and including pertussis (whooping cough), cough symptom, respiratory distress or arrest and other breathing difficulties, shortness of breath or respiratory conditions during the perinatal period).
- Fever or unspecified infection - A41.9, A49.9, A68, A99, B34.9, B97.8, B96.88, B99, R50 (fever symptom, unspecified viral infection, unspecified viraemia, unspecified bacteraemia, unspecified bacterial infection, unspecified infection).

Note: Most emergency departments used ICD-9 and SNOMED-CT codes over the period. Equivalent codes from those classification systems were used to match the ICD-10 codes above. Choice of codes was restricted in the emergency department information systems so they may not match those used for hospital admissions and deaths.
